# Enhanced echo intensity of skeletal muscle is associated with poor physical function in hemodialysis patients: a cross-sectional study

**DOI:** 10.1186/s12882-022-02816-5

**Published:** 2022-05-16

**Authors:** Junzhen Wu, Haiqing Luo, Shunrong Ren, Longxiang Shen, Dongsheng Cheng, Niansong Wang

**Affiliations:** 1 Department of Pain Management Center, Shanghai Jiao Tong University Affiliated Sixth People’s Hospital, Shanghai, China; 2 Department of Nephrology, People’s Hospital of Menghai County, Yunnan, China; 3 Department of Orthopedic Surgery, Shanghai Jiao Tong University Affiliated Sixth People’s Hospital, Shanghai, China; 4 Department of Nephrology, Shanghai Jiao Tong University Affiliated Sixth People’s Hospital, No.600, Yi Shan Road, Shanghai, 200233 P. R. China

**Keywords:** Muscle quality, Ultrasound, Physical performance, End-stage renal disease, Activities of daily living

## Abstract

**Background:**

Patients on hemodialysis often suffer from reduced muscle strength and exercise capacity due to the decreased quantity and quality of muscle. Cumulative studies showed ultrasound echo intensity (EI) had great potential in evaluating muscle quality. The objective of this study was to evaluate the relationship between EI of skeletal muscle and physical function of patients on maintenance hemodialysis.

**Methods:**

Cross-sectional area (CSA) and mean EI of the right rectus femoris were measured by ultrasound to evaluate the quantity and quality of the muscle, respectively. Physical function was measured by handgrip strength (HGS), gait speed, sit-to-stand 60 s (STS-60) test, and instrumental activities of daily living (IADL) scale.

**Results:**

A total of 107 patients on hemodialysis were included, with women accounting for 37.3% (*n* = 40), and a mean age of 53.53 ± 12.52 years. Among the patients on hemodialysis, EI was moderately and negatively correlated with HGS (*r* =  − 0.467, *P* < 0.001), gait speed (*r* =  − 0.285, *P* = 0.003), and STS-60 (*r* =  − 0.313, *P* = 0.001). Multiple regression analyses adjusted for CSA showed that the enhanced EI of patients on hemodialysis remained associated with worse HGS (β =  − 0.207, *P* = 0.047), lower gait speed (β =  − 0.002, *P* = 0.001), less STS-60 (β =  − 0.136, *P* = 0.049), and a higher likelihood of dependency in IADL (Odds Ratio: 1.070, 95% CI: [1.033–1.111], *P* = 0.001).

**Conclusions:**

In patients on hemodialysis, enhanced EI in the skeletal muscle measured via ultrasound was correlated with poor physical performance. The combined muscle quality and muscle quantity evaluation provide more information for assessing the level of physical function of the patients.

## Introduction

Patients on hemodialysis often suffer from reduced muscle strength and exercise capacity, lowering their autonomy and quality of life and shortening survival time [1]. Decreased muscle mass is one of the main causes of reduced muscle strength and exercise capacity [2, 3]. A recent study showed that muscle quality is decreased significantly at the same time as or before the decrease in muscle mass [4]. Studies of the elderly have also reported that the decline of muscle quality is closely related to the decline in physical function and poor prognosis of patients [5, 6]. Therefore, both the quantity and quality of muscle affect muscle function. The joint evaluation may help formulate intervention measures and improve prognosis.

Muscle quality is a relatively new term that refers to the microscopic and macroscopic changes in muscle structure and composition and the level of muscle function from each muscle mass unit [7]. The infiltration of non-contractile tissue such as adipose and fibrotic tissue has been the focus of numerous reports in the last decade [6, 8]. While changes in muscle composition occurred with aging, numerous factors might make chronic kidney disease (CKD) patients disposed to these alterations [9].

Multiple existing imaging methods are used to get estimated muscle composition to evaluate muscle quality. Computed tomography (CT), magnetic resonance imaging (MRI), and muscle radiation attenuation by CT evaluate muscle quality by providing the measurements of adipose tissue and lipid infiltration, loss in tissue elasticity, and relative fluid expansion [10]. Unfortunately, only a few imaging studies focused on patients on hemodialysis, possibly for the reason that CT and MRI are limited by difficulties to access due to the clinical setting, the cost, and radiation exposure. Nevertheless, a study demonstrated that CT measurement of mid-thigh intermuscular adipose tissue predicts usual gait velocity and 6-min walk distance of patients on hemodialysis [2].

Unlike MRI and CT, ultrasound has the advantages of bedside operation, convenience, non-invasiveness, and lack of radiation exposure, and is widely studied as a tool in muscle assessment. Ultrasound can effectively assess muscle mass and muscle quality with good internal and external consistency [11]. Diagnostic ultrasound has been proposed as an alternative imaging modality for the use of sarcopenia screening and staging [12]. Echo intensity (EI) is an important indicator for ultrasound assessment of muscle quality. The foundational premise of EI is that skeletal muscle is comprised not only of contractile proteins, but also non-contractile elements, such as intramuscular adipocytes and fibrous tissue [13]. Enhanced EI indicates increased infiltration of fibrotic tissue and/or adipose tissue in the muscle and represents reduced muscle quality [14]. Numerous studies in the elderly have shown that an increase in EI is related to decline in muscle strength and physical function [15–17]. A recent study involving 61 patients with CKD showed that patients with high EI had low physical function [18]. However, there are no relevant studies evaluating ultrasound derived EI in patients on hemodialysis to date. Hence, this study clarified the application value of ultrasound EI of skeletal muscle in patients on hemodialysis by assessing the relationship between ultrasound EI and physical function.

## Methods

### Study population

We performed an observational and cross-sectional study on outpatients who were on maintenance hemodialysis at Menghai county hospital (Yunnan province, China). Patients were recruited between July 2020 and August 2020, and enrolled if they had undergone more than 3 months of hemodialysis. Patients were excluded if they could not walk or be completely blind were excluded. We screened a total of 110 patients and excluded 3 patients who could not walk. A total of 107 subjects were eventually enrolled in this exploratory study. The research was conducted in accordance with the Helsinki Declaration. The patients’ demographic information, past medical history, and duration of dialysis were collected before hemodialysis. Various body measurements and assessments of physical function were performed before hemodialysis. A complete ultrasound examination of the right rectus femoris was also performed after hemodialysis.

### Body measurements and assessments of physical function

#### Measurement of body mass index (BMI)

BMI measurement of individual research subjects was calculated based on body dry weight and the following formula: BMI (Kg/m^2^) = body weight/height^2^.

#### Measurement of handgrip strength (HGS)

HGS of individual subjects was measured before hemodialysis. Patients with internal arteriovenous fistula were subjected to HGS measurement on the non-fistula hand twice to obtain the maximum value. Patients using tunneled cuffed catheters were subjected to the HGS measurement on the dominant hand twice to obtain the maximum value.

#### Gait speed

Individual subjects were required to walk independently for a 4-m distance, followed by recording of the completion time and calculation of the gait speed (meter/second) twice to obtain the maximum value.

#### Sit-to-stand 60 s (STS-60) test

The number of times an individual patient stood up and sat down on a chair within 60 s was calculated. This STS-60 test assessed the strength and endurance of muscles of the lower limbs of the tested subject [19]. The patient was asked to complete the sit down-stand up-sit down movement as many times as possible within 60 s by holding his/her hands in front of the chest and starting from the sitting position. The number of STS-60 movements was recorded at the end of the test.

#### Dependence in IADL

Physical function was also measured using the instrumental activities of daily living (IADL) scale [20, 21]. The IADL scale included 8 items: ability to shop on the street, do outdoor activities, cook meals, do household activities, wash clothes, use the telephone, take medications, and handle personal finances. The evaluation of the results of each item was divided into 3 levels: completion with no assistance, completion with partial assistance, and completion with full assistance. The IADL scale of individuals with any item which needed assistance (including partial or full assistance) was characterized as impaired IADL, otherwise IADL was characterized as intact.

### Ultrasound measurement

A portable ultrasound machine with a 60-mm width curvilinear transducer (2–5 MHz, S II, SonoSite, USA) was used to gain enough ultrasound window to cover the whole width of the rectus femoris muscle. All ultrasounds were standardized by the default machine settings (gain, and focus), and the scanning depth was set at 56 mm. One experienced pain physician who had been practicing musculoskeletal ultrasound for more than 10 years obtained all the images.

B-mode 2D ultrasonography of the right rectus femoris was taken immediately once hemodialysis was finished. The patient would lie in the supine position at a 45° angle, with the rectus femoris muscle relaxed. A line at the midpoint between the anterior superior iliac spine and the superior patellar border was marked on the skin. The ultrasound transducer was placed perpendicular to the longitudinal axis of the thigh, overlying the marked line. Minimal pressure was applied to avoid muscle distortion. In the transverse ultrasound image, the rectus femoris muscle was confirmed and the muscle epimysium boundary was identified as an oval circle wrapping around the muscle fibers. Two images from the same point for each person were saved.

**Fig. 1:**
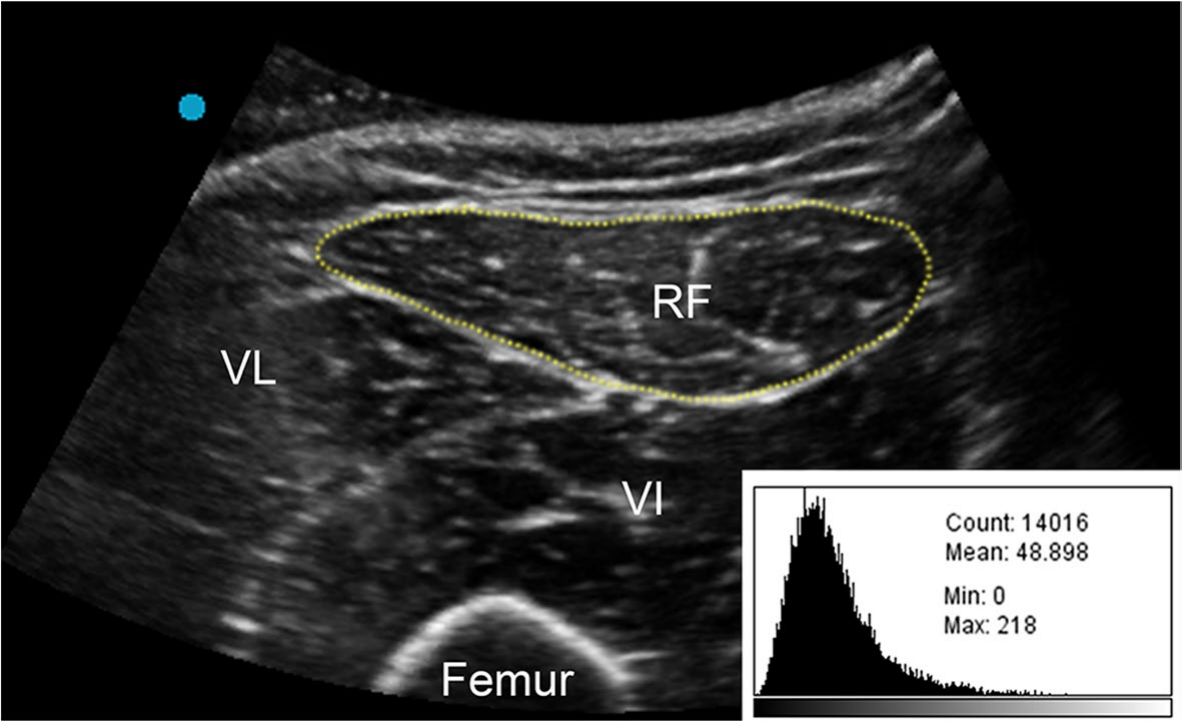
Representative ultrasound image of a relaxed rectus femoris. Rectus Femoris cross-sectional area is outlined by the dotted line. The histogram analysis for computerized quantitative grayscale analysis is illustrated at the lower right corner of the image. RF, rectus femoris; VL, vastus lateralis; VI, vastus intermedius

Ultrasound images were transferred to the computer for measurements using Image J software (National Institute of Health, USA). The whole rectus femoris muscle within its epimysium was circled as the region of interest for cross sectional area (CSA) and mean EI measurement (Fig. 1). The software returned measures of CSA and the mean EI, which was expressed as a value between 0 (black) and 255 (white) (Fig. 1). Two images in the same region for the same patient were obtained to assess test–retest reliabilities for EI values, and the average CSA and mean EI values of the two images were analyzed.

### Statistical analysis

Continuous data are presented as mean ± standard deviation, and categorical data are presented as a percentage. For continuous data, one-way ANOVA was used to compare two groups when normal distribution was fulfilled; otherwise, the non-parametric Wilcoxon rank sum test was performed. For categorical data, the chi-square test was performed. Test–retest reliability for EI values was assessed by intra-class correlation coefficient.

The association of EI with physical function was examined using Pearson’s correlation analysis. Multiple linear regression (continuous outcomes) and logistics regression (dichotomous outcomes) were performed to assess the independent association of EI and CSA with physical function. The two model methods were as follows: Model 1—only adjusted for EI and CSA; Model 2—further adjusted for gender, Model 3—further adjusted for age, gender, and dialysis duration. All statistical analyses were performed using MedCalc Software (Version 18.2.1 MedCalc Software Ltd, Belgium). *P* < 0.05 was considered statistically significant.

## Results

### Patient characteristics

As shown in Table 1, a total of 107 patients on maintenance hemodialysis were enrolled in this study. Patients were 37.3% female (*n* = 40), mean age was 53.53 ± 12.52 years, average dialysis duration was 2.97 ± 2.32 years, and average BMI was 21.43 ± 3.77 kg/m^2^. In addition, 15.9% of the patients had diabetes (*n* = 17), 93.5% of the patients had hypertension (*n* = 100), and 26.2% (*n* = 28) of the patients had dependency in IADL. The ultrasound measurements for EI and CSA are 62.34 ± 16.18 and 6.37 ± 1.58 respectively. The EI value of the male group was significantly lower than in the female group (58.21 ± 16.59 *versus* 69.26 ± 12.93, *P* < 0.001), and the CSA of the male group was significantly higher than the female group (6.91 ± 1.55 *versus* 5.46 ± 1.20, *P* < 0.001).

**Table 1:** Patient characteristics, ultrasound measurements and physical performance in patients on hemodialysis

	Total (*n* = 107)
Age (yr)	53.53 ± 12.52
BMI (kg/m^2^)	21.43 ± 3.77
EI	62.34 ± 16.18
Female	69.26 ± 12.93
Male	58.21 ± 16.59
CSA (cm^2^)	6.37 ± 1.58
Female	5.46 ± 1.20
Male	6.91 ± 1.55
Ethnicity^a^	
Han, n (%)	29 (27.1)
Dai, n (%)	41 (38.3)
Hani, n (%)	20 (18.7)
Other, n (%)	17 (15.9)
Hypertension, n (%)	100 (93.5)
Diabetes, n (%)	17 (15.9)
Primary cause of End-stage kidney disease	
Chronic glomerulonephritis, n (%)	21 (19.6)
Diabetic nephropathy, n (%)	16 (15.0)
Hypertensive nephropathy, n (%)	14 (13.1)
Obstructive nephropathy, n (%)	12 (11.2)
Other or unknown, n (%)	44 (41.1)
Dialysis duration (yr)	2.97 ± 2.32
Interdialytic weight gain (Kg)	2.90 ± 1.29
Ultrafiltration volume (L)	2.85 ± 1.19
Physical performance measures	
HGS (kg)	27.57 ± 9.49
Gait speed (m/s)	0.95 ± 0.17
STS-60 (repetitions)	29.64 ± 8.26
Dependency in IADL, n (%)	28 (26.2)

*BMI* Body mass index, *EI* Echo intensity, *CSA* Cross-sectional area, *HGS* Handgrip strength, *STS-60* Sit-to-stand 60 s, *IADL* Instrumental activities of daily living

Values are presented as mean (SD) for continuous values, and n (%) for categorical values

^a^ Ethnic background: Yunnan provinces in China had a very high level of ethnic diversity, including the Han, Dai, Hani, and so on

The intra-class correlation coefficient showed an excellent test–retest reliability for EI values (ICC = 0.948, 95% [0.925,0.965], *P* < 0.001). In addition, older age was moderated correlated with enhanced EI (*r* = 0.323, *P* = 0.001), while dialysis vintage was not correlated with EI (*r* = 0.088, *P* = 0.368).

### Relationship between muscle quality and physical function

**Fig. 2:**
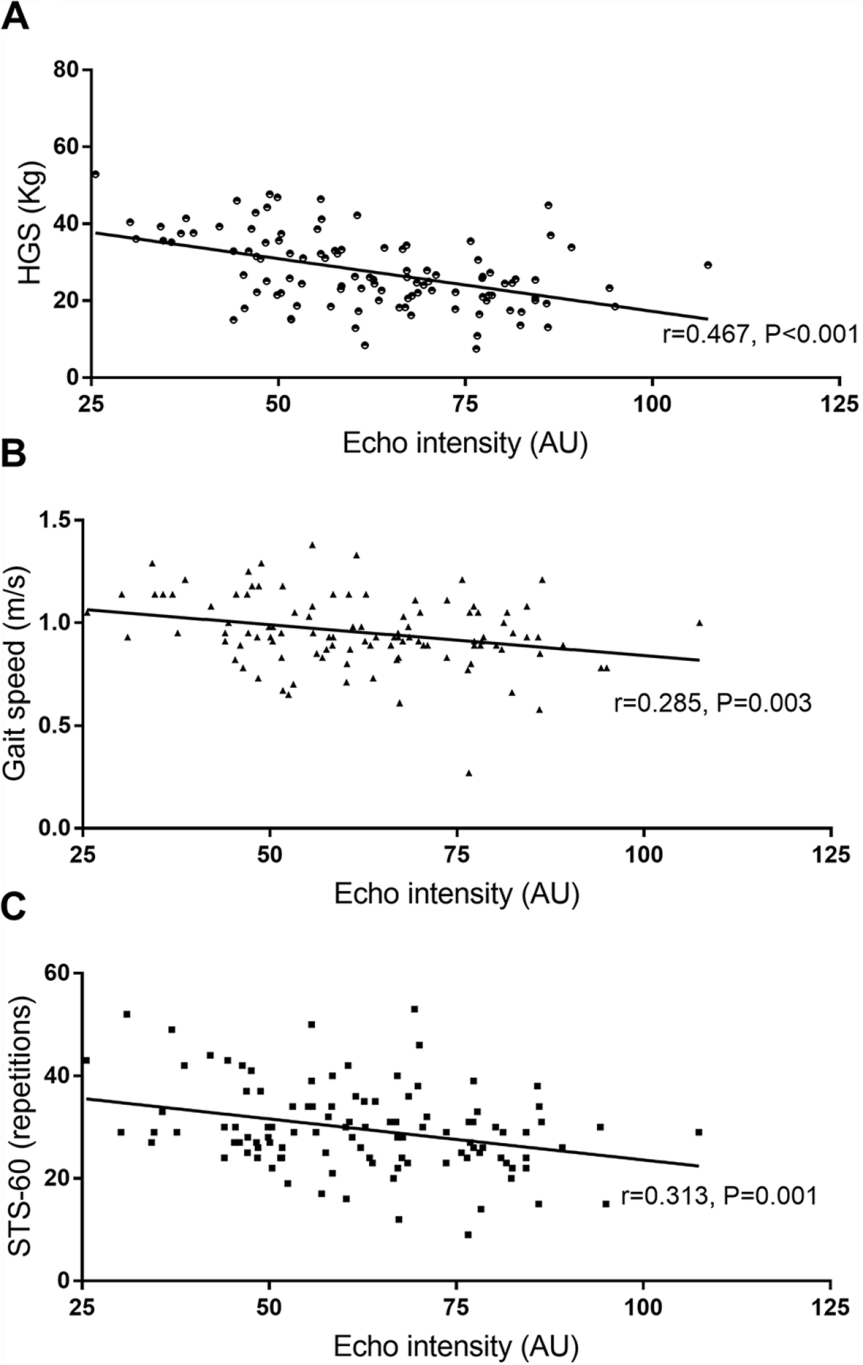
Correlation between echo intensity and physical function in all patients. a. HGS; b. Gait speed; c. STS-60. HGS, handgrip strength; STS-60, sit-to-stand 60 s

As shown in Fig. 2, the EI of the overall patient population on hemodialysis was negatively correlated with HGS (*r* =  − 0.467, *P* < 0.001), gait speed (*r* =  − 0.285, *P* = 0.003), and STS-60 (*r* =  − 0.313, *P* = 0.001).

### Comparison of physical function with muscle quality (EI) and muscle quantity (CSA)

Further multiple linear regression analyses and logistic regression analyses were performed to assess the relationship between EI, CSA, and physical function. As shown in Table 2, Model 1 adjusted for the EI and CSA, Model 2 further adjusted for gender, and Model 3 further adjusted for age, gender, and dialysis duration. Enhanced EI was associated with worse HGS (β =  − 0.207, *P* = 0.047), lower gait speed (β =  − 0.002, *P* = 0.001), less STS-60 (β =  − 0.136, *P* = 0.049), and a higher likelihood of dependency in IADL in Model 1 (OR: 1.070; 95% CI: [1.030–1.111], *P* = 0.001). These associations remained statistically significant after further adjusting for sex in Model 2, but enhanced EI was only associated with dependency in IALD (P = 0.001) in Model 3. Except for STS-60, increased CSA was a significant predictor of better HGS, higher gait speed, and lower IALD disability among all Model 1, Model 2, and Model 3 in patients on hemodialysis.

**Table 2:** Comparisons of physical function with muscle quality (EI) and muscle quantity (CSA) in patients on hemodialysis

	Model 1		Model 2		Model 3	
	β (SE)	*P-value*	β (SE)	*P-value*	β (SE)	*P-value*
HGS (kg)						
EI	− 0.207 (0.047)	< 0.001	− 0.171 (0.046)	< 0.001	− 0.090 (0.047)	0.061
CSA	2.527 (0.479)	< 0.001	1.912 (0.500)	< 0.001	1.393 (0.483)	0.005
Gait speed (m/s)						
EI	− 0.002 (0.001)	0.028	− 0.002 (0.001)	0.017	− 0.002 (0.001)	0.130
CSA	0.030 (0.010)	0.003	0.035 (0.011)	0.002	0.030 (0.011)	0.009
STS-60 (repetitions)						
EI	− 0.136 (0.049)	0.006	− 0.150 (0.050)	0.003	− 0.081 (0.051)	0.119
CSA	0.892 (0.497)	0.075	1.131 (0.539)	0.038	0.747 (0.522)	0.156
	Odds ratio (95% CI)	*P-value*	Odds ratio (95% CI)	*P-value*	Odds ratio (95% CI)	*P-value*
Dependency in IADL						
EI	1.070 (1.030–1.1112)	0.001	1.100 (1.049–1.155)	< 0.001	1.086 (1.033–1.141)	0.001
CSA	0.569 (0.376–0.860)	0.008	0.369 (0.216–0.633)	< 0.001	0.438 (0.255–0.755)	0.003

Multiple linear regression for continuous outcomes and logistic regression for dichotomous outcomes. Model 1: EI + CSA; Model 2: EI + CSA + sex; Model 3: EI + CSA + sex + age + dialysis duration

*HGS* Handgrip strength, *EI* Echo intensity, *CSA* Cross-sectional area, *STS-60* Sit-to-stand 60 s, *IADL* Instrumental activities of daily living

## Discussion

This study was the first to report the relationship between ultrasound-derived EI of the skeletal muscle and physical function in patients on hemodialysis. Our results showed that high EI was closely related to low physical function including HGS, gait speed, STS-60, and IADL disability in patients on hemodialysis.

The pathophysiological mechanisms of decreased muscle quality include muscle fiber reduction, mitochondrial dysfunction of myocytes, intramuscular fat infiltration, and increased fibrosis [22]. The existing assessment methods of muscle quality mainly include CT, MRI, and ultrasound. Although CT and MRI directly evaluate the degree of intramuscular fat infiltration, they are expensive and require patients to go to a specific place or have a certain degree of radiation, which affects patient compliance with the examination. Ultrasound is being increasingly implemented by investigators in the fields of muscle measurements, taking ultrasound EI as a vital indicator of muscle quality. Compared with percent intramuscular fat measurements derived from high-resolution T1-weighted MRI imaging, ultrasound EI proved to be a practical and reproducible method that can be used as an imaging technique for examination of percent intramuscular fat [23]. Age-related increases in EI, which represents decreases in muscle quality with increasing intramuscular adipose and fibrous tissue, have been reported in numerous studies. A comparison study demonstrated that the young group had significantly lower EI than that of the older group, and there are significant and moderate correlations between EI and CT values (*r* = -0.502) and between EI and percentage of low-density muscle area (*r* = 0.441), suggesting that higher EI at least partly reflects intramuscular lipid infiltration [24].

Numerous studies in the elderly have confirmed the close relationship between ultrasound-derived EI isometric muscle strength and physical function. In elderly men, the ultrasound EI showed a significant negative correlation with muscle strength (*r* =  − 0.333), and multivariate regression analysis revealed that the EI of the knee extensor muscle was independently associated with maximum isometric knee extension strength [16]. A study on volunteer elder men resulted that quadriceps femoris EI was correlated to functional capacity and power countermovement jump, and the maximal number of repetitions completed in the 30-s sit-to-stand test presented weak to moderate significant correlation with EI of four quadriceps components [17]. In a study, participants were categorized into the low, mid, and high EI groups according to the EI of the thigh. The researchers found that the high EI group exhibited a significantly slower 5-m walking time and shorter 6-min walking distance than the low EI group and had a shorter moderate-intensity activity time than the mid EI group [25]. In the field of kidney diseases, only one small-scale study of stages 3–5 CKD pre-dialysis patients has reported that EI is moderately and negatively correlated with the STS-60 test, the incremental shuttle walk test (ISWT) (*n* = 61), and peak oxygen consumption (VO_2_ peak) results (*n* = 32), but not with the gait speed and HGS (*n* = 29); EI was not correlated with any index of physical function when the CSA was corrected [18]. In this study, EI was moderately and negatively correlated with HGS, gait speed, and STS-60 in patients on hemodialysis. Moreover, after adjusting for muscle area, high EI in patients on hemodialysis remained associated with worse HGS, low gait speed, reduced STS-60, and IADL disability. Thus, this is the first report showing the value of ultrasound-derived EI in muscle evaluation of patients on hemodialysis.

As expected, analysis of the ultrasound parameters of the rectus femoris showed that the CSA of the male group was significantly higher than in the female group, and the EI value of the male group was markedly lower than in the female group. Male muscles are stronger, with larger CSA of the rectus femoris muscle. These phenomena are consistent with other studies. A CT imaging study investigating skeletal muscle quantity and quality in end-stage renal disease showed that muscle CSA was significantly greater in men than in women [2]. The proportion of body fat in the females was generally higher than that of males, and then, the EI value of the female skeletal muscle was higher. A number of studies have reported that EI values are greater in women than in men in both young and old adult populations [13]. In addition, our study lighted that after adjusting for sex in Model 2 of multiple linear regression analyses and logistic regression, enhanced EI remained associated with worse HGS, lower gait speed, less STS-60, and a higher likelihood of dependency in IADL. Therefore, the relationship between physical function and EI (and CSA) is likely to be independent to sex. This result was just consistent with previous studies [10, 26].

In addition to muscle quality, muscle quantity is also an essential indicator for muscle evaluation of patients. A wealth of evidence from multiple studies has shown that muscle size is closely related to the physical function of patients on hemodialysis [2, 3, 27]. When we further corrected for factors such as age, gender, and the duration of dialysis, only CSA was related to the level of physical function of the patients. Thus, in the clinical setting, the combined evaluation of muscle quantity and muscle quality may provide more information for accurately assessing muscle strength and physical function in patients [10].

Loss of muscle quantity and quality in patients on hemodialysis may be related to increased oxidative stress, accumulation of uremic toxins, reduced exercise activities, and malnutrition [28, 29]. Exercise training is an effective way to improve muscle function. In patients with CKD, ultrasound measurement of CSA has been used to effectively assesses muscle mass gain after exercise intervention [30]. Impendence movement reduced the accumulation of fat in the muscles of the elderly [31]. However, there are quite a few researches on the improvement of muscle quality through exercise in patients on hemodialysis. A recent study revealed that seven months of intradialytic aerobic exercise training improved functional capacity and prevented thigh muscle mass loss in hemodialysis patients, and indicated that muscle ultrasonography could play a pivotal role in assessing muscle quality changes in hemodialysis patients [32]. However, the researchers used the vastus lateralis fascicle angle as the ultrasound measurement of the muscle quality in that study. Indeed, further research is needed to verify whether EI can be used as an evaluation index for the improvement of muscle quality.

One of the concerns about the validity of muscle echogenicity as a surrogate measure of muscle quality in dialysis patients is that ultrasound technology is relatively less established and highly tester-and analyzer-dependent by nature. Unfortunately, so far no consistent protocol has arrived for image acquisition and analysis in the field of muscle measurements. EI values may differ between different studies and are not comparable. In our study, we applied some methods to make the results reliable. First, we used a portable ultrasound device in our study, and all the parameters (gain, focus, dynamic range and frame rate) were kept in the default settings of the machine, with the scanning depth at 56 mm. We didn’t change these parameters during the whole process of measurement for the worry that any change of the parameters would complicate the comparison of the data obtained from different patients. Second, the CSA and mean EI values of the two images obtained in the same region for the same patient were averaged to analyze to minimize the instability of ultrasound probe held by the operator. Last, considering the effects of fluid status on ultrasound muscle measurement in patients on hemodialysis, we took ultrasonography of the right rectus femoris after hemodialysis process. Ultrasound-based evaluation of muscle quantity and quality of patients on hemodialysis provided more information for assessing the level of physical function in patients. It has significant clinical value and broad application prospects in the muscle evaluation of patients on hemodialysis. Nevertheless, there is an urgent need to develop a consensus on scanning technique and image analysis for ultrasound muscle measurement.

This study had some limitations: First, as no standardized measurement method for EI is currently available, we used the raw data of EI. Subcutaneous adipose thickness can attenuate ultrasound waves and influence muscle EI reliability; an additional method in ultrasound muscle quality measurement is to adjust EI [13]. However, besides depth, the transmission of ultrasound beams through the tissue can be attenuated due to complicated factors, such as reflection, dispersion, or absorption of the sound waves. Second, we measured regional leg muscles to estimate systemic muscle quality in patients on hemodialysis, the relation of EI of leg muscle with other limb functionality may be reserved, and further studies need to clarify the relationship. Third, this was a cross-sectional study, so it is impossible to clarify the etiological inference of the decline in muscle quality represented by EI and the decreased physical function of patients on hemodialysis. In addition, this study was carried out in a single dialysis center in China, and the generalization of our conclusions requires international, multi-center studies.

## Conclusion

The present study revealed that enhanced ultrasound-derived EI was correlated with poor physical performance in patients on hemodialysis. The combined muscle quality and muscle quantity evaluation provides more information for assessing the level of physical function of the patients.

## Data Availability

All data generated or analysed during this study are included in this published article.

## Abbreviations

EI: Echo intensity
CSA: Cross-sectional area
HGS: Handgrip strength
STS-60: Gait speed, sit-to-stand 60 s
IADL: Instrumental activities of daily living
CT: Computed tomography; MRI: magnetic resonance imaging
CKD: Chronic kidney disease
BMI: Body mass index
ISWT: Incremental shuttle walk test
SD: Standard deviation
SE: Standard error
